# Prevalence of liver steatosis and stiffness by transient elastography and serum markers in Japanese early adolescents

**DOI:** 10.1265/ehpm.26-00150

**Published:** 2026-10-20

**Authors:** Ayaka Ito, Yuki Ito, Sayaka Kato, Yukihiro Ohya, Daiki Hiraoka, Kyoko Minato, Gui Yang, Shigeaki Hida, Mayumi Sugiura-Ogasawara, Shinji Saitoh, Michihiro Kamijima

**Affiliations:** 1 Department of Occupational and Environmental Health, Nagoya City University Graduate School of Medical Sciences, 1 Kawasumi, Mizuho-cho, Mizuho-ku, Nagoya 467-8601, Japan; 2 Department of Molecular and Cellular Health Sciences, Graduate School of Pharmaceutical Sciences, Nagoya City University, Nagoya 467-8603, Japan; 3 Department of Obstetrics and Gynecology, Nagoya City University Graduate School of Medical Sciences, 1 Kawasumi, Mizuho-cho, Mizuho-ku, Nagoya 467-8601, Japan; 4 Department of Pediatrics and Neonatology, Nagoya City University Graduate School of Medical Sciences, 1 Kawasumi, Mizuho-cho, Mizuho-ku, Nagoya 467-8601, Japan

**Keywords:** Child, Controlled attenuation parameter (CAP), Hepatic steatosis, Liver steatosis, Liver stiffness measurement (LSM), Metabolic dysfunction–associated steatotic liver disease (MASLD), School-aged children

## Abstract

**Background:**

The prevalence of metabolic dysfunction–associated steatotic liver disease (MASLD) is projected to increase in the Asian pediatric population. However, data regarding its prevalence in the general pediatric population are limited. We aimed to determine the prevalence of liver steatosis and liver stiffness, measured by controlled attenuation parameter (CAP) and liver stiffness measurement (LSM), respectively, using FibroScan^®^ in the general pediatric population, and to assess their associations with sex, BMI, abdominal circumference (AC), and serum parameters.

**Methods:**

The CAP and LSM were measured in 1,435 children aged 9–12 years in the Aichi Regional Adjunct Study of the Japan Environment and Children’s Study. Elevated CAP and LSM values were defined by age-specific CAP and LSM cutoffs (11–12 and 9–10 years: CAP >225 and >220 dB/m and LSM >6.5 and >5.8 kPa, respectively). Multivariate logistic regression, adjusted for sex, BMI, AC, HDL-Chol, non-HDL-Chol, TG, and ALT levels, was performed.

**Results:**

At 11–12 years, the prevalence of elevated CAP and LSM was 12.6% and 2.9%, respectively. Children with higher CAP exhibited significantly higher BMI and AC. TG and ALT levels were significantly associated with higher CAP, whereas lower non-HDL-Chol levels were significantly associated with higher LSM. At 9–10 years, higher LSM was more prevalent in males.

**Conclusion:**

This study reports the prevalence of elevated CAP and LSM values and their association with biomarkers in a large cohort of the general pediatric population in Japan. This partially contributes to the understanding of the epidemiology of MASLD and supports the development of early prevention and risk-stratification approaches.

**Supplementary information:**

The online version contains supplementary material available at https://doi.org/10.1265/ehpm.26-00150.

## Graphical Abstract

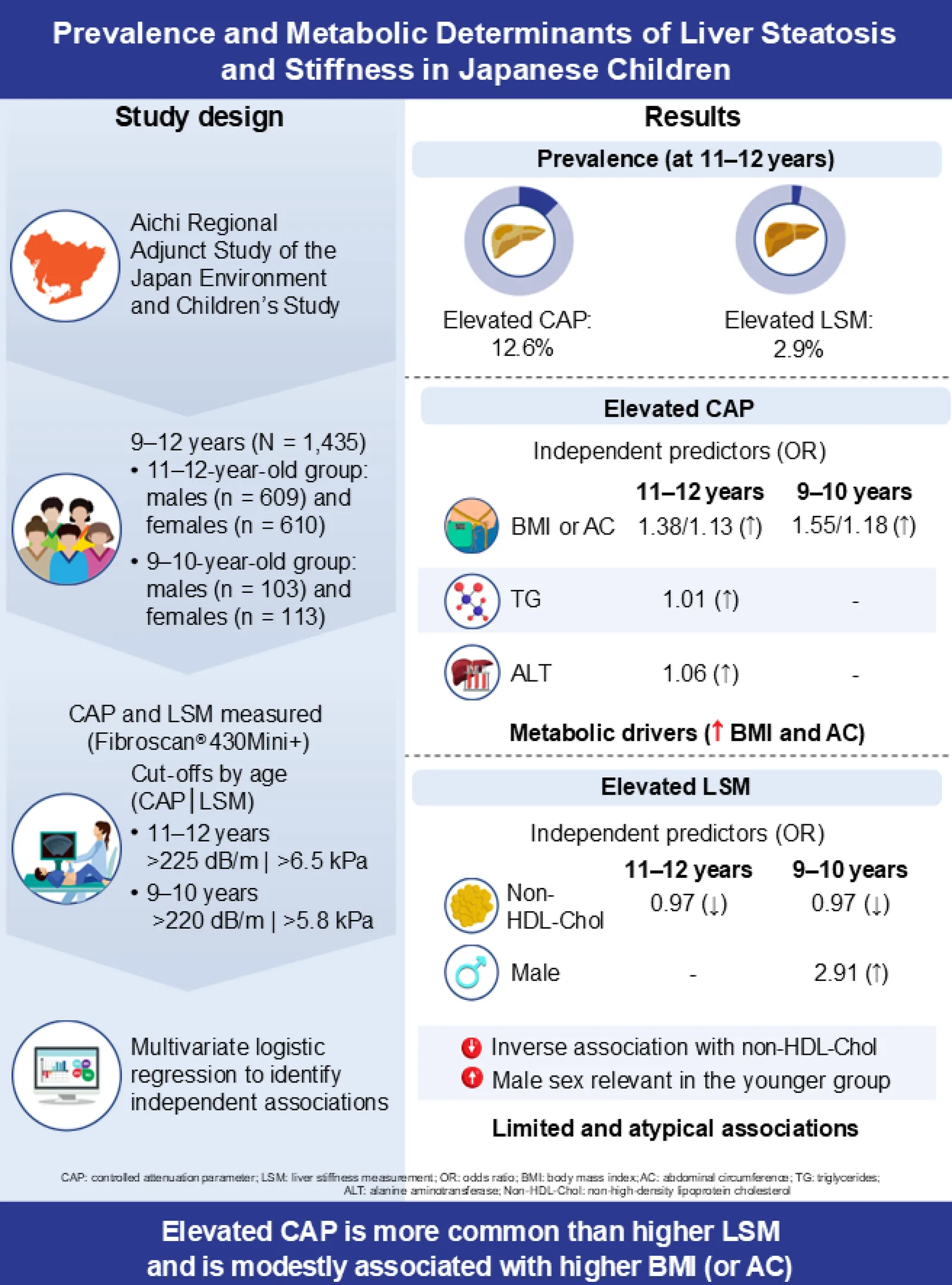

## 1. Background

The prevalence of metabolic dysfunction–associated steatotic liver disease (MASLD), formerly known as non-alcoholic fatty liver disease (NAFLD), in the general pediatric population has increased in recent years. Recent reviews have estimated a global MASLD prevalence of 7–14% in children [1, 2], which increases up to 38.0%, particularly in those with obesity [1]. In Eastern Asia, the prevalence of fatty liver among children with obesity was 50% in a cohort of 186 children aged <16 years [3]. In a recent Shanghai study, the prevalence of MASLD was 3.42% and 15.93% in healthy 8-year-olds and children with obesity, respectively [4], and is projected to increase sharply [2]. MASLD constitutes a known risk factor for several metabolic diseases. Steatotic liver disease (SLD) in children is associated with hepatic inflammation, and may progress to MASLD, metabolic dysfunction–associated steatohepatitis, cirrhosis, or metabolic syndrome in adulthood [5]. Therefore, early screening for MASLD in childhood and adolescence is crucial to enable early intervention and reduce the risk of long-term complications [2].

The gold standard for diagnosing SLD is histopathological examination of liver biopsy specimens. However, liver biopsy is highly invasive and requires general anesthesia in children, which is not feasible for epidemiological studies. Recently, substantial attention has been directed toward vibration-controlled transient elastography (VCTE) using FibroScan^®^ (Echosens, Paris, France) because it is a noninvasive, painless, examiner-independent, rapid, and reproducible method for assessing liver steatosis and fibrosis. FibroScan^®^ provides two parameters: controlled attenuation parameter (CAP) for detecting hepatic steatosis using ultrasound signals and shear wave propagation-based liver stiffness measurement (LSM) for assessing liver fibrosis. The feasibility of this method in the general pediatric population has been demonstrated in children, including preschoolers [6, 7] and very young children [7].

Liver steatosis and stiffness, measured by CAP and LSM, respectively, using FibroScan^®^ are associated with age, sex, and various biochemical parameters in the general pediatric population [4, 8–10]. However, evidence of this phenomenon in Asia remains limited. Worldwide, no study has examined the associations of liver steatosis and stiffness with serum biochemical markers in healthy elementary school children.

We aimed to determine CAP and LSM values to estimate the prevalence of liver steatosis and stiffness in a large cohort of the general pediatric population in Japan and to examine the associations between elevated CAP and LSM values and age, sex, body size, and serum biochemical indices. The cohort comprised approximately 40% of the population of the study area [11], and this enhanced the data representativeness and robustness.

## 2. Methods

### 2.1 Participants

This Adjunct Study of the Aichi Regional Cohort of the Japan Environment and Children’s Study (JECS), a nationwide prospective birth cohort study in Japan, investigated environmental factors affecting children’s health and development [11–13]. Eligible participants were children undergoing health checkups in the JECS Main or Sub-Cohort Study in a Regional Cohort. A health checkup in the JECS Main Study was conducted at ages 11–12 years, whereas that in the Sub-Cohort Study was conducted at ages 9–10 years in approximately 5% of randomly selected JECS-main participants [14]. During a face-to-face health checkup, the legal guardians of children in the Aichi Regional Cohort of the JECS (JECS-A) provided written informed consent to allow their children to participate in this study. All examinations were conducted after obtaining assent from the children during the checkup. Of the 2,703 children in the target population, 1,209 who did not participate in face-to-face health checkups during the period and 38 who did not agree with or complete FibroScan^®^ measurements were excluded (Additional File 1: Fig. S1). Furthermore, 21 participants whose data did not meet the reliability criteria were excluded. A total of 1,435 children were included: 609 males and 610 females aged 11–12 years and 103 males and 113 females aged 9–10 years participated in face-to-face health checkups from July 2023 to October 2024 and October 2023 to August 2024, respectively.

The JECS protocol was reviewed and approved by the Institutional Review Board for Epidemiological Studies of the Japanese Ministry of the Environment (approval no. 100910001). The study protocol was approved by the Institutional Review Board of the Nagoya City University Graduate School of Medical Sciences (approval no. 70-19-0001). This study was conducted in accordance with the principles of the Declaration of Helsinki and applicable national regulations and guidelines.

### 2.2 FibroScan^®^

Liver steatosis and stiffness were assessed using CAP and LSM obtained by VCTE with the FibroScan^®^ 430Mini+ system equipped with Smart Exam functions. Measurements were performed using the M probe in accordance with the manufacturer’s instructions, and all examiners were trained and certified by Echosens, Inc. Briefly, the participants were instructed to fast for at least 2 h and lie in a supine position with the left side slightly flexed in a banana shape. The examiners placed the probe transducer on the right rib cage. LSM was considered reliable when based on at least 10 valid measurements, with the median value used for analysis and an interquartile range (IQR)–to–median ratio ≤30%. A mean CAP level >100% was used for a statistical analysis when the standard deviation (SD) was ≤30.

### 2.3 Height, body weight (BW), and abdominal circumference (AC)

Body height, BW, and AC were measured in the standing position by trained medical staff. Height was measured twice using a portable stadiometer (Seca 213l, Chiba, Japan), and the mean value was used for the analysis. The AC was measured twice using a tape measure. Differences >1 cm between two measurements of height or AC warranted a third measurement, and the mean of the two closest values was used for the analysis. BW was measured once using a body-composition monitor (MC-780A; Tanita, Tokyo, Japan). Participants were required to wear light clothing with a standard clothing weight of 0.5 kg applied. The BMI standard deviation scores (BMI-SDS) were calculated using the LMS method [15], reported by the Japanese Society for Pediatric Endocrinology.

### 2.4 Blood samples

Blood samples were collected during the medical interviews. With legal guardian consent, 18 mg lidocaine patches (Nitto, Tokyo, Japan) were applied to the planned puncture sites at least 30 min before blood collection. Serum was collected and used to measure HDL-Chol, T-Chol, TG, and ALT levels using Spotchem D-3 (ARKRAY, Inc., Kyoto, Japan). Non-HDL-Chol was calculated as T-Chol minus HDL-Chol.

### 2.5 Statistical analysis

Participants were analyzed separately in two age groups: 11–12 and 9–10 years. In participants aged 11–12 years, we used a cutoff of CAP >225 dB/m and LSM >6.5 kPa to define elevated CAP and elevated LSM values suggestive of liver steatosis and advanced fibrosis, respectively [16]. Participants aged 9–10 years were dichotomized according to the 90th percentile of the CAP and LSM. Fasting status was assessed by calculating the time interval between the last meal and the FibroScan measurement, as fasting durations of 15–45 min affect measurements [17]. A Gibbs sampler–based left-censored missing value-imputation approach (GSimp) [18] was used to impute values below the lower limit of detection of ALT. For the other missing values, R package “mice” was used for multiple imputation by chained equation, and 20 datasets were pooled and used for further analyses. Continuous variables are expressed as means and SD or as medians and IQR. Categorical variables are expressed as counts and percentages. Between-group comparisons were performed using Welch’s two-sample *t*-test and Fisher’s exact test for continuous and categorical variables, respectively. Univariate and multivariate logistic regression analyses were performed to analyze the associations between the binarized CAP, LSM, and clinical variables. Missing values were handled using multiple imputations prior to logistic regression. Univariate logistic regression was used to assess the associations between sex, height, BW, BMI, AC, AC/height, HDL-Chol, non-HDL-Chol, TG, and ALT. Multivariate models were adjusted for sex, BMI, AC, and HDL-Chol, non-HDL-Chol, TG, and ALT levels. For participants aged 9–10 years, the LSM models were adjusted for sex, BMI, AC, non-HDL-Chol, and ALT levels. For sensitivity analyses, data without multiple imputations were analyzed using logistic regression. Statistical significance was set at *p* < 0.05. Data analyses were performed using R software (version 4.4.1).

## 3. Results

Table 1 summarizes the characteristics of the 1,435 participants. The mean age and proportion of males in the 11–12- and 9–10-year-old groups were 11.8 ± 0.28 and 10.1 ± 0.31 years and 50% (n = 609) and 48% (n = 103), respectively; significant between-group differences in height, BW, AC, and levels of HDL-Chol and in mean CAP and LSM (184 vs. 176 dB/m and 4.32 vs. 4.59 kPa, respectively) were observed.

**Table 1:** Participant characteristics.

**Characteristic**	**11–12 years, n = 1,219**		**9–10 years, n = 216**		***p* value**
	**(n)**		**(n)**		
Age (year)	1219	11.8 (0.28)	216	10.1 (0.31)	**<0.01**
Sex	1219		216		
Male		609 (50%)		103 (48%)	0.56
Female		610 (50%)		113 (52%)	
Height (cm)	1213	149 (7)	216	138 (6)	**<0.01**
BW (kg)	1215	40 (8)	216	32 (7)	**<0.01**
BMI-SDS	1212	−0.29 (1.10)	216	−0.24 (1.07)	0.49
AC (cm)	1218	65 (8)	216	61 (8)	**<0.01**
AC/Height	1212	0.44 (0.05)	216	0.44 (0.05)	0.37
HDL-Chol (mg/dL)	1004	58 (15)	177	65 (18)	**<0.01**
Non-HDL-Chol (mg/dL)	1004	110 (22)	177	107 (22)	0.13
T-Chol (mg/dL)	1004	168 (23)	177	171 (24)	0.0502
TG (mg/dL)	1004	79 [57–110]	177	74 [53–103]	0.12
ALT (IU/L)	1004	13 [10–17]	177	13 [11–17]	0.81
Time from last meal to FibroScan (h)	1219		216		
<0.75		2 (0.2%)		5 (2.3%)	**<0.01**
0.75–<1		3 (0.2%)		8 (3.7%)	
≥1		1,214 (99.6%)		203 (94%)	

Sex is reported as count (%), TG and ALT as median [IQR], and other values as mean (SD). One participant in the Main Study who drank a sweetened soft drink 0.85 h before the FibroScan measurement was included in the variable, “Time from last meal to FibroScan” (≤1 h). When the time from last meal to blood collection was also calculated, significant differences in the p value were noted (*p* < 0.01, 11–12 years: 4.3% [<2 h], 95.7% [≥2 h], and 9–10 years: 11% [<2 h], 89% [≥2 h]).

Mean CAP significantly differed in boys and girls (189 ± 40 vs. 178 ± 34 dB/m; *p* < 0.01) in the 11–12-year-old group, but not in the 9–10-year-old group (179 ± 34 vs. 172 ± 36 dB/m; *p* = 0.16). In the 11–12 and 9–10-year-old groups, mean LSM was similar between boys and girls (4.34 ± 0.96 vs. 4.31 ± 1.00 kPa; *p* = 0.54 and 4.71 ± 1.46 vs. 4.48 ± 0.97 kPa; *p* = 0.17, respectively).

Table 2 presents a comparison of participants above and below the CAP cutoff value. In the 11–12-year-old group, 12.6% (n = 154) of the participants had elevated CAP values (>225 dB/m). Participants with elevated CAP had significantly higher BW, BMI-SDS, AC, AC/height, non-HDL-Chol, TG, and ALT levels, but lower HDL-Chol levels, than those without elevated CAP. The proportion of males was higher in the elevated CAP value group than in the other groups. No other characteristics differed between the two groups. In the 9–10-year-old group, 10.2% (n = 22) of the participants had elevated CAP values (>220 dB/m) and showed significantly higher BW, BMI-SDS, AC, and AC/height than those with CAP values below the cutoff, with no other significant between-group differences.

**Table 2:** Characteristics stratified by CAP value (dB/m).

**Characteristic**	**11–12 years**					**9–10 years**				
	
	**CAP > 225 dB/m** **n = 154**		**CAP ≤ 225 dB/m** **n = 1,065**		***p* value**	**CAP > 220 dB/m** **n = 22**		**CAP ≤ 220 dB/m** **n = 194**		***p* value**
	**(n)**		**(n)**			**(n)**		**(n)**		
Age (year)	154	11.8 (0.29)	1065	11.8 (0.28)	0.22	22	10.0 (0.28)	194	10.1 (0.32)	0.69
Sex	154		1065			22				
Male		94 (61%)		515 (48%)	**<0.01**		11 (50%)	194	92 (47%)	0.83
Female		60 (39%)		550 (52%)			11 (50%)		102 (53%)	
Height (cm)	153	149 (7)	1060	149 (7)	0.12	22	140.3 (6.3)	194	137.6 (5.9)	0.06
BW (kg)	154	47 (10)	1061	39 (7)	**<0.01**	22	40.9 (10.6)	194	31.3 (5.3)	**<0.01**
BMI-SDS	152	0.69 (1.13)	1060	−0.43 (1.03)	**<0.01**	22	0.76 (1.57)	194	−0.35 (0.94)	**<0.01**
AC (cm)	154	75 (11)	1064	63 (7)	**<0.01**	22	72 (12)	194	59 (6)	**<0.01**
AC/Height	153	0.50 (0.07)	1059	0.43 (0.04)	**<0.01**	22	0.51 (0.09)	194	0.43 (0.04)	**<0.01**
HDL-Chol (mg/dL)	122	54 (16)	882	58 (14)	**<0.01**	15	64 (23)	162	65 (17)	0.87
Non-HDL-Chol (mg/dl)	122	117 (23)	882	109 (21)	**<0.01**	15	111 (27)	162	107 (21)	0.56
T-Chol (mg/dL)	122	171 (23)	882	167 (22)	0.06	15	175 (24)	162	171 (24)	0.61
TG (mg/dL)	122	106 [73–162]	882	75 [55–105]	**<0.01**	15	78 [53–161]	162	74 [53–101]	0.35
ALT (IU/L)	122	18 [13–27]	882	13 [<10–16]	**<0.01**	15	15 [12–20]	162	13 [10–17]	0.11
Time from last meal to FibroScan (h)	164		1055			22		194		
<0.75		1 (0.6%)		1 (<0.1%)	0.27		1 (4.5%)		4 (2.1%)	0.39
0.75–<1		0 (0%)		3 (0.3%)			1 (4.5%)		7 (3.6%)	
≥1		153 (99.4%)		1,061 (99.7%)			20 (91%)		183 (94%)	

Sex is reported as count (%), TG and ALT as median [IQR], and other values as mean (SD). One participant in the CAP ≤ 225 dB/m group of 11–12 years, who drank a sweetened soft drink 0.85 h before the FibroScan measurement, was included in the group of “≤1 h” Time from last meal to FibroScan. As for the time from last meals to blood collection, no significant differences were observed (11–12 years: *p* = 0.35; CAP > 225 dB/m, 2.3% [<2 h]; 97.7% [≥2 h] vs CAP ≤ 225 dB/m, 4.6% [<2 h]; 95.4% [≥2 h]; 9–10 years: *p* = 0.20; CAP > 220 dB/m, 20% [<2 h]; 80% [≥2 h] vs CAP ≤ 220 dB/m, 9.8% [<2 h]; 90.2% [≥2 h])

Table 3 presents a comparison of the participants above and below the LSM cutoff values. In the 11–12-year-old group, 2.9% (n = 35) of the participants had elevated LSM values (LSM >6.5 kPa). Participants with higher LSM values had significantly lower non-HDL-Chol and T-Chol, whereas HDL-Chol levels did not differ from those without increased stiffness; there were no other between-group differences. In the 9–10-year-old group, 9.3% (n = 20) of the participants had elevated LSM values (>5.8 kPa). Participants with higher LSM values had significantly lower non-HDL-Chol, whereas HDL-Chol and T-Chol levels did not differ, compared with those in the ≤90th percentile group. When the same cutoff values used for the 11–12-year-old group were applied to the 9–10-year-old group, elevated CAP and elevated LSM values were observed in 9.3% (n = 11) and 3.7% (n = 8), respectively. Univariate logistic regression (Table 4) revealed that, in the 11–12-year-old group, male sex (odds ratio [OR], 1.67; 95% confidence interval [CI], 1.18–2.36) and per-unit increases in BMI (1.49; 1.39–1.58), AC (1.15; 1.13–1.18), non-HDL-Chol (1.02; 1.01–1.03), TG (1.01; 1.007–1.013), and ALT (1.10; 1.07–1.12) were significantly associated with higher CAP values and per-unit decrease in HDL-Chol (0.98; 0.96–0.992) was significantly associated with higher CAP values. In the 9–10-year-old group, per-unit increases in BMI (1.55; 1.31–1.84), AC (1.18; 1.11–1.26), and ALT (1.05; 1.01–1.09) were significantly associated with higher CAP values. Regarding LSM, a per-unit increase in non-HDL-Chol (0.97; 0.95–0.992) was inversely associated with higher LSM values. At 9–10 years, male sex (2.81; 1.03–7.64) and non-HDL-Chol (0.97; 0.95–0.997) were significantly associated with higher LSM values. The multivariable logistic regression analysis (Tables 5 [model including BMI] and 6 [model including AC instead of BMI]) revealed that, in the 11–12-year-old group, per-unit increases in BMI (or AC) (BMI: 1.38; 1.28–1.49; AC: 1.13; 1.10–1.16), TG (BMI and AC: 1.01; 1.004–1.01), and ALT (1.06 or 1.05; 1.03–1.08) were significantly associated with higher CAP values. In the 9–10-year-old group, BMI (or AC) (BMI: 1.55; 1.28–1.87; AC: 1.18; 1.11–1.26), but not TG and ALT, was significantly associated with higher CAP values. Regarding LSM, non-HDL-Chol (BMI and AC: 0.97; BMI: 0.95–0.989 or AC: 0.95–0.990) was inversely associated with higher LSM values at 11–12 years, and, at 9–10 years, male sex (BMI model: 2.91; 1.01–8.35; AC model: 2.89; 1.01–8.28), and non-HDL-Chol (0.97; 0.94–0.9994 or 0.9991) were significantly associated with higher LSM values. These results are comparable to those obtained without multiple imputations using T-Chol instead of non-HDL-Chol (Additional File 1: Tables S1–S3).

**Table 3:** Characteristics stratified by LSM value (kPa).

**Characteristic**	**11, 12 years**					**9, 10 years**				
	
	**LSM > 6.5 kPa** **n = 35**		**LSM ≤ 6.5 kPa** **n = 1,184**		***p* value**	**LSM > 5.8 kPa** **n = 20**		**LSM ≤ 5.8 kPa** **n = 196**		***p* value**
	**(n)**		**(n)**			**(n)**		**(n)**		
Age (year)	35	11.9 (0.26)	1184	11.8 (0.28)	0.01	20	10.0 (0.33)	196	10.1 (0.31)	0.40
Sex	35		1184		>0.99	20		196		0.06
Male		17 (49%)		592 (50%)			14 (70%)		89 (45%)	
Female		18 (51%)		592 (50%)			6 (30%)		107 (55%)	
Height (cm)	35	150 (7)	1178	149 (7)	0.29	20	138.3 (7.8)	196	137.8 (5.8)	0.80
BW (kg)	35	42 (8)	1180	40 (8)	0.14	20	31.7 (5.0)	196	32.3 (6.8)	0.59
BMI-SDS	35	−0.10 (1.17)	1177	−0.30 (1.10)	0.33	20	−0.30 (0.94)	196	−0.23 (1.09)	0.76
AC (cm)	35	66 (9)	1183	65 (8)	0.42	20	60 (6)	196	61 (8)	0.68
AC/Height	35	0.44 (0.06)	1177	0.44 (0.05)	0.62	20	0.43 (0.04)	196	0.44 (0.05)	0.56
HDL-Chol (mg/dL)	27	58 (16)	977	58 (15)	0.87	15	69 (22)	162	64 (18)	0.42
Non-HDL-Chol (mg/dl)	27	97 (19)	977	110 (22)	**<0.01**	15	95 (23)	162	108 (21)	**0.04**
T-Chol (mg/dL)	27	155 (26)	977	168 (22)	**0.01**	15	164 (27)	162	172 (24)	0.24
TG (mg/dL)	27	62 [46–90]	977	79 [57–111]	0.14	15	62 [38–103]	162	75 [56–103]	0.29
ALT (IU/L)	27	13 [<10–19]	977	13 [10–17]	0.37	15	12 [<10–16]	162	14 [11–17]	0.06
Time from last meal to FibroScan (h)	39		1180			20		196		
<0.75		0 (0%)		2 (0.2%)	>0.99		1 (3.8%)		4 (2.1%)	0.49
0.75–<1		0 (0%)		3 (0.3%)			0 (0%)		8 (4.2%)	
≥1		35 (100%)		1,179 (99.5%)			19 (96.2%)		184 (93.7%)	

Sex is shown as number (%), TG and ALT as median [IQR], and other values as mean (SD). One participant in the LSM ≤ 6.5 kPa group of 11–12 years, who drank a sweetened soft drink 0.85 h before the FibroScan measurement, was included in the “≥1 h” Time from last meal to FibroScan. As for the time from last meal to blood collection, no significant differences were observed (11–12 years: *p* > 0.999; LSM > 6.5 kPa, 3.3% [<2 h]; 96.7% [≥2 h], vs. LSM ≤ 6.5 kPa, 4.3% [<2 h]; 95.7% [≥2 h]. 9–10 years: *p* > 0.999; LSM > 5.8 kPa, 11% [<2 h]; 89% [≥2 h], vs. LSM ≤ 5.8 kPa, 11% [<2 h]; 89% [≥2 h]).

**Table 4:** Univariate logistic regression for higher CAP (a) and LSM (b) in children after multiple imputations.

a) CAP					

**Characteristic**		**n**	**OR**	**95% CI**	***p* value**
11, 12 years	Sex	1219			
	Male		**1.67**	**1.18, 2.36**	**<0.01**
	BMI	1219	**1.49**	**1.39, 1.58**	**<0.01**
	AC (cm)	1219	**1.15**	**1.13, 1.18**	**<0.01**
	HDL-Chol (mg/dL)	1219	**0.98**	**0.96, 0.992**	**<0.01**
	Non-HDL-Chol (mg/dL)	1219	**1.02**	**1.01, 1.03**	**<0.01**
	TG (mg/dL)	1219	**1.01**	**1.007, 1.013**	**<0.01**
	ALT (IU/L)	1219	**1.10**	**1.07, 1.12**	**<0.01**
9, 10 years	Sex	216			
	Male		1.11	0.46, 2.69	0.82
	BMI	216	**1.55**	**1.31, 1.84**	**<0.01**
	AC (cm)	216	**1.18**	**1.11, 1.26**	**<0.01**
	HDL-Chol (mg/dL)	216	0.99	0.96, 1.02	0.62
	Non-HDL-Chol (mg/dL)	216	1.01	0.98, 1.03	0.51
	TG (mg/dL)	216	1.00	0.997, 1.01	0.22
	ALT (IU/L)	216	**1.05**	**1.01, 1.09**	**0.02**

b) LSM					

**Characteristic**		**n**	**OR**	**95% CI**	***p* value**
11, 12 years	Sex	1219			
	Male		0.94	0.48, 1.85	0.87
	BMI	1219	1.08	0.97, 1.19	0.17
	AC (cm)	1219	1.02	0.98, 1.06	0.40
	HDL-Chol (mg/dL)	1219	0.99	0.97, 1.02	0.62
	Non-HDL-Chol (mg/dL)	1219	**0.97**	**0.95, 0.992**	**<0.01**
	TG (mg/dL)	1219	0.99	0.98, 1.004	0.25
	ALT (IU/L)	1219	1.00	0.97, 1.03	0.88
9, 10 years	Sex	216			
	Male		**2.81**	**1.03, 7.64**	**0.04**
	BMI	216	0.94	0.78, 1.13	0.51
	AC (cm)	216	0.99	0.93, 1.06	0.74
	HDL-Chol (mg/dL)	216	1.01	0.98, 1.04	0.51
	Non-HDL-Chol (mg/dL)	216	**0.97**	**0.95, 0.997**	**0.03**
	TG (mg/dL)	216	0.99	0.98, 1.01	0.43
	ALT (IU/L)	216	0.94	0.86, 1.04	0.21

The multiple imputation methods GSimp and mice were used for values below the lower limit of detection for ALT and other missing values, respectively.

**Table 5:** Multivariable regression for higher CAP (a) and LSM (b) in children after multiple imputations.

a) CAP						

**Characteristic**		**n**	**OR**	**95% CI**	**p value**	**VIF**
11, 12 years	Sex	1219				
	Male		0.97	0.62, 1.51	0.89	1.17
	BMI	1219	**1.38**	**1.28, 1.49**	**<0.01**	1.24
	HDL-Chol (mg/dL)	1219	1.01	0.996, 1.03	0.13	1.29
	Non-HDL-Chol (mg/dL)	1219	1.00	0.987, 1.01	0.68	1.27
	TG (mg/dL)	1219	**1.01**	**1.004, 1.01**	**<0.01**	1.19
	ALT (IU/L)	1219	**1.06**	**1.03, 1.08**	**<0.01**	1.21
9, 10 years	Sex	216				
	Male		0.79	0.26, 2.36	0.67	1.04
	BMI	216	**1.55**	**1.28, 1.87**	**<0.01**	1.14
	HDL-Chol (mg/dL)	216	1.02	0.98, 1.06	0.29	1.45
	Non-HDL-Chol (mg/dL)	216	1.00	0.97, 1.03	0.93	1.26
	TG (mg/dL)	216	1.00	0.992, 1.01	0.63	1.25
	ALT (IU/L)	216	1.03	0.997, 1.07	0.07	1.09

b) LSM						

**Characteristic**		**n**	**OR**	**95% CI**	***p* value**	**VIF**
11, 12 years	Sex	1219				
	Male		0.96	0.46, 2.01	0.92	1.1
	BMI	1219	1.14	0.988, 1.30	0.07	1.36
	HDL-Chol (mg/dL)	1219	0.99	0.96, 1.01	0.33	1.20
	Non-HDL-Chol (mg/dL)	1219	**0.97**	**0.95, 0.989**	**<0.01**	1.21
	TG (mg/dL)	1219	1	0.988, 1.01	0.50	1.22
	ALT (IU/L)	1219	0.99	0.95, 1.03	0.72	1.33
9, 10 years	Sex	216				
	Male		**2.91**	**1.01, 8.35**	**0.048**	1.03
	BMI	216	1.01	0.81, 1.25	0.94	1.05
	Non-HDL-Chol (mg/dL)	216	**0.97**	**0.94, 0.9994**	**0.045**	1.07
	ALT (IU/L)	216	0.91	0.81, 1.02	0.11	1.13

The multiple imputation methods GSimp and mice were used for values below the lower limit of detection for ALT and other missing values, respectively.

**Table 6:** Multivariable regression for higher CAP and LSM with AC replacing BMI, after multiple imputations.

a) CAP						

**Characteristic**		**n**	**OR**	**95% CI**	***p* value**	**VIF**
11, 12 years	Sex	1219				
	Male		0.81	0.52, 1.27	0.36	1.17
	AC (cm)	1219	**1.13**	**1.10, 1.16**	**<0.01**	1.30
	HDL-Chol (mg/dL)	1219	1.01	0.998, 1.03	0.09	1.28
	Non-HDL-Chol (mg/dL)	1219	1.00	0.986, 1.01	0.58	1.28
	TG (mg/dL)	1219	**1.01**	**1.004, 1.01**	**<0.01**	1.18
	ALT (IU/L)	1219	**1.05**	**1.03, 1.08**	**<0.01**	1.21
9, 10 years	Sex	216				
	Male		0.60	0.19, 1.88	0.38	1.07
	AC (cm)	216	**1.18**	**1.11, 1.26**	**<0.01**	1.16
	HDL-Chol (mg/dL)	216	1.02	0.98, 1.06	0.30	1.47
	Non-HDL-Chol (mg/dL)	216	1.00	0.97, 1.03	0.83	1.28
	TG (mg/dL)	216	1.00	0.991, 1.01	0.75	1.25
	ALT (IU/L)	177	1.03	0.994, 1.07	0.14	1.09

b) LSM						

**Characteristic**		**n**	**OR**	**95%CI**	***p* value**	**VIF**
11, 12 years	Sex	1219				
	Male		0.90	0.44, 1.86	0.78	1.09
	AC (cm)	1219	1.04	0.98, 1.09	0.19	1.43
	HDL-Chol (mg/dL)	1219	0.98	0.96, 1.01	0.29	1.19
	Non-HDL-Chol (mg/dL)	1219	**0.97**	**0.95, 0.990**	**<0.01**	1.21
	TG (mg/dL)	1219	1.00	0.988, 1.01	0.51	1.22
	ALT (IU/L)	1219	1.00	0.94, 1.03	0.80	1.35
9, 10 years	Sex	216				
	Male		**2.89**	**1.01, 8.28**	**0.048**	1.03
	AC (cm)	216	1.01	0.93, 1.08	0.88	1.05
	Non-HDL-Chol (mg/dL)	216	**0.97**	**0.94, 0.9991**	**0.04**	1.06
	ALT (IU/L)	216	0.91	0.81, 1.02	0.11	1.13

The multiple imputation methods GSimp and mice were used for values below the lower limit of detection for ALT and other missing values, respectively.

## 4. Discussion

To our knowledge, this is the first study to investigate the prevalence and characteristics of liver steatosis and stiffness using FibroScan^®^ in a large cohort of the general pediatric population in Japan. Among those aged 11–12 years, 12.6% (154/1219) had elevated CAP values, and higher BMI (or AC) was associated with higher CAP. In contrast, 2.9% (35/1219) of participants had elevated LSM values, and higher non-HDL-Chol levels were inversely associated with higher LSM.

The prevalence of elevated CAP values varies across studies from China and the US, possibly owing to differences in cutoff values. Zeng et al. reported a prevalence of fatty liver disease of 5.07% among 848 general Chinese children aged 8 years [4]. Ciardullo et al. reported a prevalence of liver steatosis of 24.2% among 867 participants aged 12–18 years in the National Health and Nutrition Examination Survey (NHANES) [19]. Although we defined CAP >225 dB/m as suggestive of liver steatosis in the 11–12-year-old group, the prevalence decreased to 5.3% in the same population with a CAP cutoff value >248 dB/m [20], which implied fat accumulation in at least 5% of the liver cells, as used in the Chinese and American reports. In contrast, a study using the same cutoff value reported a prevalence of 21.7% in 60 pediatric patients with juvenile idiopathic arthritis receiving methotrexate therapy [21], which was higher than that observed in our study. However, reports involving 139–482 participants in Germany aged 10–18 years [9], Japanese children with obesity aged 1–18 years [22], and adolescents in the NHANES aged 12–19 years [23] showed that CAP did not differ among the investigated age groups. This contrasts with our results, in which the mean CAP observed in children aged 11–12 years was higher than in children aged 9–10 years. This difference may be explained by the higher abdominal obesity and BMI in older children.

The prevalence of elevated CAP values was substantially higher among males than females aged 11–12 years, a finding consistent with those of most previous studies in adolescents [10, 19]. Furthermore, the prevalence of MASLD is markedly higher in males than in females in both adult and pediatric populations [1, 10]. This sex difference may be partly explained by the protective effects of estrogen, as evidenced by data showing that premenopausal women have a lower prevalence of MASLD than postmenopausal women [25]. Interactions between insulin resistance, fat-deposition differences, cytokine and adipokine profiles, and sex hormones may contribute to sex differences in MASLD in the youth [26]. However, in the present study, no significant difference in CAP was observed between boys and girls aged 9–10 years. The prevalence of MASLD did not differ between boys and girls aged 8 years [4]. Further investigations are required to clarify the effects of increasing age.

Participants with elevated CAP values had substantially higher BW, BMI, and AC than those without increased steatosis, which is consistent with previous reports showing that higher CAP was positively associated with BMI [19], BMI above the 90th percentile [27], or BMI-SDS [9]. In another study, the CAP values showed notable positive correlations with BMI, height, BW, and waist circumference [4]. Moreover, CAP values were higher in individuals with obesity than in healthy participants [4, 27]. Obesity is a major risk factor for MASLD [1, 28, 29]. Obesity could interact with NAFLD, leading to the deterioration of metabolic status in 6,086 Chinese children aged 6–18 years [29]. Together, these findings suggest that body size is an important determinant of liver steatosis.

Participants with elevated CAP values at 11–12 years had markedly higher TG and ALT values in both the crude and adjusted models, higher non-HDL-Chol only in the crude model, and lower HDL-Chol only in the adjusted model compared with children with unelevated CAP values. Few studies have examined the associations between serum parameters and liver steatosis in the general pediatric population. However, two studies on adolescents from the NHANES support our findings. Participants aged 12–18 years with higher CAP values had substantially higher TG and ALT levels and lower HDL-Chol [19], whereas those with SLD had markedly higher ALT levels [30].

The prevalence of elevated LSM values in 11–12-year-old children was 2.9%, which was lower than the 5.9% reported in the US, despite our use of a lower cutoff for liver stiffness (LSM >6.5 kPa) than the higher cutoff (LSM >7.4 kPa) used to define significant fibrosis in the US [19]. This may be partly because only 0.4% of participants exceeded the adult reference value of 8.2 kPa [31]. This proportion was lower than that in the other groups, which suggests that severe cases were included in the other groups.

Elevated liver stiffness, expressed as higher LSM values, was significantly associated with male sex in the group aged 9–10 years, whereas the mean LSM did not differ significantly between the sexes in the overall cohort. The LSM was relatively stable between 10 and 18 years of age and was higher in boys [9]. In another study, aging was associated with higher LSM among children aged ≥3 years, whereas male sex was not associated with an increase in LSM [8]. Thus, sex differences in LSM values were inconsistent across studies.

Notably, children aged 11–12 and 9–10 years with liver stiffness had markedly lower non-HDL-Chol levels, whereas previous studies have reported associations between higher T-Chol levels and MASLD [24, 28]. Although MASLD is commonly associated with obesity, it can also occur in the absence of obesity, and non-obese MASLD has become increasingly common. The prevalence of non-obese MASLD among adults was 38% in East Asia [29]. The reason for the inverse association between higher LSM and lower non-HDL-Chol remains unclear. Because the number of participants with values above the cutoff was limited, the findings should be interpreted with caution, and a longitudinal evaluation of this relationship is warranted in future studies.

A major strength of this study was its large scale. Children in the general population have few opportunities to undergo blood tests or ultrasonography. However, the availability of general pediatric FibroScan data is limited. Although CAP and LSM are adjunctive rather than definitive diagnostic indicators, our findings may contribute to the development of biochemical markers to detect early-stage MASLD worldwide and provide a basis for future international epidemiological studies on pediatric liver health.

This study has some limitations. First, the number of participants in the 9–10-year-old group was limited. Selection bias cannot be ruled out because the eligible participants represented approximately half of the total cohort in the 11–12-year-old group, which potentially limits the generalizability of our findings. Second, a direct comparison of the prevalence between the 11–12- and 9–10-year-old groups was not possible because different cutoffs for CAP and LSM were used. The cutoff of the present study was not an externally validated criterion, but rather, a provisional working definition borrowed from another cohort [16]. Further studies are necessary to establish age-dependent references and cutoff values for liver steatosis and stiffness using FibroScan. Third, the present findings primarily describe liver steatosis assessed using FibroScan rather than MASLD itself because we did not evaluate metabolic dysfunction according to the current MASLD definition. Finally, because a dry chemistry analyzer was used in this study, which differs from chemistry analyzers used in clinical practice, caution is required when applying these measurements to clinical settings.

## 5. Conclusions

This study demonstrated that the prevalence of elevated CAP and LSM values was 12.6% and 2.9%, respectively, in a large cohort of Japanese early adolescents aged 11–12 years. Higher BMI (or AC) was associated with increased CAP values in the general pediatric population. These findings may inform the development of MASLD-preventive strategies in children.

## Abbreviations

AC: abdominal circumference
ALT: alanine aminotransferase
BMI: body mass index
BMI-SDS: body mass index-standard deviation scores
BW: body weight
CAP: controlled attenuation parameter
Cl: confidence interval
GSimp: Gibbs sampler based left-censored missing value imputation approach
HDL-Chol: high-density lipoprotein cholesterol
IQR: interquartile range
IQR/Median: interquartile range to the median
JECS: the Japan Environment and Children’s Study
JECS-A: the Aichi Regional Cohort of JECS
LSM: liver stiffness measurement
MASLD: metabolic dysfunction-associated steatotic liver disease
NHANES: National Health and Nutrition Examination Survey
non-HDL-Chol: non-high-density lipoprotein cholesterol
OR: odds ratio
SD: standard deviation
SDS: standard deviation scores
SLD: steatotic liver disease
T-Chol: total cholesterol
TG: triglyceride
VCTE: vibration-controlled transient elastography
VIF: variance inflation factor

## Supplementary information

**Additional file 1:** **Table S1.** Univariate logistic regression for higher CAP (a) and LSM (b) in children without multiple imputations. **Table S2.** Multivariable regression for higher CAP (a) and LSM (b) in children without multiple imputations. **Table S3.** Multivariable regression for higher CAP and LSM with AC replacing BMI, without multiple imputations. **Figure S1.** Participant flow diagram. **Figure S2.** Age-related distributions of LSM and CAP scores. **Figure S3.** Scatterplots showing the relationships between CAP/LSM and height, weight, BMI-SDS, AC, AC/Height, HDL-Chol, non-HDL-Chol, T-Chol, TG, and ALT levels. a) CAP, b) LSM, i. 11–12-year-old group. ii. 9–10-year-old group.

## Declarations

### Availability of data and materials

The data and materials used to derive our conclusions are not suitable for public deposition. The Act on the Protection of Personal Information (Act No. 57 of May 30, 2003, amended on September 9, 2015) prohibits public deposition of data containing personal information. The Ethical Guidelines for Epidemiological Research enforced by the Japan Ministry of Education, Culture, Sports, Science, and Technology and the Ministry of Health, Labor, and Welfare also restrict the open sharing of epidemiological data.
