# Supplementary material for: Prevalence of liver steatosis and stiffness by transient elastography and serum markers in Japanese early adolescents
Source: Environ Health Prev Med. 2026 Oct 20;31:70. doi: 10.1265/ehpm.26-00150 (PMC13719248; doi:10.1265/ehpm.26-00150)
Supplement: Supplementary file 1 [file ehpm-31-070-s001.docx]

**Supplementary Information**

**Prevalence of liver steatosis and stiffness by transient elastography and serum markers in Japanese early adolescents**

Ayaka Ito^1^, Yuki Ito^1^, Sayaka Kato^1^, Yukihiro Ohya^1^, Daiki Hiraoka^1^, Kyoko Minato^1^, Gui Yang^1^, Shigeaki Hida^2^, Mayumi Sugiura-Ogasawara^3^, Shinji Saitoh^4^, Michihiro Kamijima^1^

1Department of Occupational and Environmental Health, Nagoya City University Graduate School of Medical Sciences, 1 Kawasumi, Mizuho-cho, Mizuho-ku, Nagoya, 467-8601, Japan

2Department of Molecular and Cellular Health Sciences, Graduate School of Pharmaceutical Sciences, Nagoya City University, Nagoya, 467-8603, Japan

3Department of Obstetrics and Gynecology, Nagoya City University Graduate School of Medical Sciences, 1 Kawasumi, Mizuho-cho, Mizuho-ku, Nagoya, 467-8601, Japan

4Department of Pediatrics and Neonatology, Nagoya City University Graduate School of Medical Sciences, 1 Kawasumi, Mizuho-cho, Mizuho-ku, Nagoya, 467-8601, Japan

*Correspondence: [yukey@med.nagoya-cu.ac.jp](mailto:yukey@med.nagoya-cu.ac.jp) (YI); [kamijima@med.nagoya-cu.ac.jp](mailto:kamijima@med.nagoya-cu.ac.jp) (MK)

**Table S1.** Univariate logistic regression for higher CAP (a) and LSM (b) in children without multiple imputations.

**Table S2.** Multivariable regression for higher CAP (a) and LSM (b) in children without multiple imputations.

**Table S3.** Multivariable regression for higher CAP and LSM with AC replacing BMI, without multiple imputations.

**Figure S1.** Participant flow diagram

**Figure S2.** Age-related distributions of LSM and CAP scores

**Figure S3.** Scatter plots showing the relationships between CAP/LSM and height, BW, BMI-SDS, AC, AC/Height, HDL-Chol, non-HDL-Chol, T-Chol, TG, and ALT levels. a) CAP, b) LSM, i. 11–12-year-old group. ii. 9–10-year-old group. Orange, Female; Blue, Male.

**Table S1.** Univariate logistic regression for higher CAP (a) and LSM (b) in children without multiple imputations.

a)	CAP

| Characteristic | | n | OR | 95% CI | *p* value |
| --- | --- | --- | --- | --- | --- |
| 11, 12 years | Sex | 1219 |  |  |  |
|  | Male |  | **1.67** | 1.19, 2.37 | **<0.01** |
|  | BMI | 1212 | **1.48** | 1.39, 1.58 | **<0.01** |
|  | AC (cm) | 1218 | **1.15** | 1.13, 1.18 | **<0.01** |
|  | HDL-Chol (mg/dL) | 1004 | **0.98** | 0.97, 0.99 | **<0.01** |
|  | T-Chol (mg/dL) | 1004 | 1.01 | 1.000, 1.02 | 0.06 |
|  | TG (mg/dL) | 1004 | **1.01** | 1.007, 1.012 | **<0.01** |
|  | ALT (IU/L) | 1004 | **1.09** | 1.06, 1.11 | **<0.01** |
| 9, 10 years | Sex | 216 |  |  |  |
|  | Male |  | 1.11 | 0.45, 2.71 | 0.82 |
|  | BMI | 216 | **1.55** | 1.33, 1.87 | **<0.01** |
|  | AC (cm) | 216 | **1.18** | 1.12, 1.26 | **<0.01** |
|  | HDL-Chol (mg/dL) | 177 | 1.00 | 0.97, 1.03 | 0.83 |
|  | T-Chol (mg/dL) | 177 | 1.01 | 0.98, 1.03 | 0.61 |
|  | TG (mg/dL) | 177 | 1.00 | 0.996, 1.01 | 0.27 |
|  | ALT (IU/L) | 177 | **1.04** | 1.003, 1.08 | **0.04** |

b)	LSM

| Characteristic | | n | OR | 95% CI | *p* value |
| --- | --- | --- | --- | --- | --- |
| 11, 12 years | Sex | 1219 |  |  |  |
|  | Male |  | 0.94 | 0.48, 1.86 | 0.87 |
|  | BMI | 1212 | 1.08 | 0.96, 1.19 | 0.17 |
|  | AC (cm) | 1218 | 1.02 | 0.98, 1.05 | 0.40 |
|  | HDL-Chol (mg/dL) | 1004 | 1.00 | 0.97, 1.02 | 0.87 |
|  | T-Chol (mg/dL) | 1004 | **0.97** | 0.95, 0.99 | **<0.01** |
|  | TG (mg/dL) | 1004 | 0.99 | 0.98, 1.002 | 0.19 |
|  | ALT (IU/L) | 1004 | 0.99 | 0.94, 1.02 | 0.59 |
| 9, 10 years | Sex | 216 |  |  |  |
|  | Male |  | **2.81** | 1.08, 8.20 | **0.04** |
|  | BMI | 216 | 0.94 | 0.76, 1.11 | 0.51 |
|  | AC (cm) | 216 | 0.99 | 0.92, 1.05 | 0.74 |
|  | HDL-Chol (mg/dL) | 177 | 1.01 | 0.99, 1.04 | 0.31 |
|  | T-Chol (mg/dL) | 177 | 0.99 | 0.96, 1.01 | 0.19 |
|  | TG (mg/dL) | 177 | 0.99 | 0.98, 1.005 | 0.35 |
|  | ALT (IU/L) | 177 | 0.93 | 0.84, 1.01 | 0.13 |

The average of ten repeated GSimp measurements was used for values below the lower limit of detection for ALT.

**Table S2.** Multivariable regression for higher CAP (a) and LSM (b) in children without multiple imputations.

a) CAP

| Characteristic | | n | OR | 95% CI | p value | VIF |
| --- | --- | --- | --- | --- | --- | --- |
| 11, 12 years | Sex | 1219 |  |  |  |  |
|  | Male |  | 0.90 | 0.56, 1.45 | 0.66 | 1.18 |
|  | BMI | 1212 | **1.34** | 1.24, 1.46 | **<0.01** | 1.27 |
|  | HDL-Chol (mg/dL) | 1004 | 1.01 | 0.995, 1.03 | 0.15 | 1.58 |
|  | T-Chol (mg/dL) | 1004 | 1.00 | 0.99, 1.01 | 0.98 | 1.29 |
|  | TG (mg/dL) | 1004 | **1.01** | 1.004, 1.01 | **<0.01** | 1.18 |
|  | ALT (IU/L) | 1004 | **1.05** | 1.03, 1.08 | **<0.01** | 1.24 |
| 9, 10 years | Sex | 216 |  |  |  |  |
|  | Male |  | 0.54 | 0.15, 1.77 | 0.32 | 1.03 |
|  | BMI | 216 | **1.44** | 1.19, 1.80 | **<0.01** | 1.11 |
|  | HDL-Chol (mg/dL) | 177 | 1.02 | 0.98, 1.06 | 0.34 | 1.65 |
|  | T-Chol (mg/dL) | 177 | 1.00 | 0.97, 1.03 | 0.85 | 1.27 |
|  | TG (mg/dL) | 177 | 1.00 | 0.99, 1.01 | 0.64 | 1.27 |
|  | ALT (IU/L) | 177 | 1.03 | 0.99, 1.07 | 0.09 | 1.10 |

b) LSM

| Characteristic | | n | OR | 95% CI | *p* value | VIF |
| --- | --- | --- | --- | --- | --- | --- |
| 11, 12 years | Sex | 1219 |  |  |  |  |
|  | Male |  | 0.82 | 0.36, 1.85 | 0.63 | 1.11 |
|  | BMI | 1212 | 1.09 | 0.92, 1.27 | 0.31 | 1.26 |
|  | HDL-Chol (mg/dL) | 1004 | 1.02 | 0.99, 1.05 | 0.21 | 1.53 |
|  | T-Chol (mg/dL) | 1004 | **0.97** | 0.94, 0.99 | **<0.01** | 1.37 |
|  | TG (mg/dL) | 1004 | 1.00 | 0.99, 1.01 | 0.57 | 1.18 |
|  | ALT (IU/L) | 1004 | 0.99 | 0.94, 1.02 | 0.71 | 1.24 |
| 9, 10 years | Sex | 216 |  |  |  |  |
|  | Male |  | **3.78** | 1.19, 14.6 | **0.03** | 1.05 |
|  | BMI | 216 | 0.97 | 0.73, 1.24 | 0.83 | 1.09 |
|  | T-Chol (mg/dL) | 177 | 0.98 | 0.96, 1.01 | 0.15 | 1.82 |
|  | ALT (IU/L) | 177 | 0.90 | 0.81, 0.996 | 0.05 | 1.25 |

The average of ten repeated GSimp measurements was used for values below the lower limit of detection for ALT.

**Table S3.** Multivariable regression for higher CAP and LSM with AC replacing BMI, without multiple imputations.

a) CAP

| Characteristic | | n | OR | 95% CI | *p* value | VIF |
| --- | --- | --- | --- | --- | --- | --- |
| 11, 12 years | Sex | 1219 |  |  |  |  |
|  | Male |  | 0.72 | 0.44, 1.16 | 0.18 | 1.19 |
|  | AC (cm) | 1218 | **1.12** | 1.09, 1.16 | **<0.01** | 1.31 |
|  | HDL-Chol (mg/dL) | 1004 | 1.02 | 0.998, 1.03 | 0.08 | 1.59 |
|  | T-Chol (mg/dL) | 1004 | 1.00 | 0.99, 1.01 | 0.83 | 1.31 |
|  | TG (mg/dL) | 1004 | **1.01** | 1.004, 1.01 | **<0.01** | 1.18 |
|  | ALT (IU/L) | 1004 | **1.05** | 1.02, 1.08 | **<0.01** | 1.24 |
| 9, 10 years | Sex | 216 |  |  |  |  |
|  | Male |  | 0.43 | 0.11, 1.48 | 0.20 | 1.07 |
|  | AC (cm) | 216 | **1.16** | 1.08, 1.25 | **<0.01** | 1.14 |
|  | HDL-Chol (mg/dL) | 177 | 1.02 | 0.98, 1.06 | 0.37 | 1.56 |
|  | T-Chol (mg/dL) | 177 | 1.01 | 0.98, 1.03 | 0.74 | 1.22 |
|  | TG (mg/dL) | 177 | 1.00 | 0.99, 1.01 | 0.75 | 1.28 |
|  | ALT (IU/L) | 177 | 1.03 | 0.99, 1.07 | 0.14 | 1.09 |

b) LSM

| **Characteristic** | | n | OR | 95%CI | *p* value | VIF |
| --- | --- | --- | --- | --- | --- | --- |
| 11, 12 years | Sex | 1219 |  |  |  |  |
|  | Male |  | 0.78 | 0.34, 1.76 | 0.56 | 1.09 |
|  | AC (cm) | 1218 | 1.02 | 0.96, 1.08 | 0.48 | 1.29 |
|  | HDL-Chol (mg/dL) | 1004 | 1.02 | 0.99, 1.05 | 0.24 | 1.53 |
|  | T-Chol (mg/dL) | 1004 | **0.97** | 0.94, 0.99 | **<0.01** | 1.37 |
|  | TG (mg/dL) | 1004 | 1.00 | 0.99, 1.01 | 0.57 | 1.18 |
|  | ALT (IU/L) | 1004 | 0.99 | 0.94, 1.02 | 0.76 | 1.24 |
| 9, 10 years | Sex | 216 |  |  |  |  |
|  | Male |  | **3.82** | 1.21, 14.7 | **0.03** | 1.03 |
|  | AC (cm) | 216 | 0.98 | 0.88, 1.08 | 0.75 | 1.05 |
|  | T-Chol (mg/dL) | 177 | 0.98 | 0.96, 1.01 | 0.15 | 1.82 |
|  | ALT (IU/L) | 177 | 0.9 | 0.81, 0.996 | 0.05 | 1.22 |

The average of ten repeated GSimp measurements was used for values below the lower limit of detection for ALT.

Main Study (ages 11–12 years)

Sub-Cohort Study (ages 9–10 years)

n = 2703

Participated in face-to-face health checkup

n = 1456

Not participated

n = 1209

Total n = 1435

11–12 years	n = 1219 (609 males and 610 females)

9–10 years	n = 216 (103 males and 113 females)

Not meet reliability criteria

n = 21

No agreement or not completed measurement

n = 38

**Figure S1.** Participant flow diagram

a)	CAP

i.	The 11–12-year-old group

ii.	The 9‒10-year-old group

b)	LSM

i.	The 11–12-year-old group

ii.	The 9‒10-year-old group

**Figure S2.** Age-related distributions of LSM and CAP scores

a) CAP
i. 11–12-year-old group
ii. 9‒10-year-old group

b)	LSM

i.	11–12-year-old group

ii.	9‒10-year-old group

**Figure S3.** Scatterplots showing the relationships between CAP/LSM and height, BW, BMI-SDS, AC, AC/Height, HDL-Chol, non-HDL-Chol, T-Chol, TG, and ALT levels. a) CAP, b) LSM, i. 11–12-year-old group. ii. 9–10-year-old group. Orange, Female; Blue, Male. A Gibbs sampler–based left-censored missing value-imputation approach (GSimp) was used to impute values <10 IU/L of ALT. The trends in the plots remained unchanged after imputing ALT values below 10 IU/L.
